# The use of transcutaneous electrical acupoint stimulation to reduce opioid consumption in patients undergoing off-pump CABG: a randomized controlled trial

**DOI:** 10.1186/s13741-024-00427-2

**Published:** 2024-07-05

**Authors:** Hui Zhang, Lini Wang, Ziyu Zheng, Jiange Han, Lin Li, Wenlong Yao, Zhijian Li, Gang Luo, Baobao Gao, Jie Shen, Hailong Dong, Chong Lei

**Affiliations:** 1 Department of Anesthesiology and Perioperative Medicine, Xijing Hospital, Fourth Military Medical University, Xi’an, 710032 China; 2 Department of Anesthesiology, Tianjin Chest Hospital, Tianjin, China; 3 Department of Anesthesiology, General Hospital of Northern Theater Command, Shenyang, China; 4 Department of Anesthesiology, Tongji Hospital, Tongji Medical College, Huazhong University of Science and Technology, Wuhan, China; 5 Department of Anesthesiology, The Second Xiangya Hospital, Central South University, Changsha, China

**Keywords:** Transcutaneous electrical acupoint stimulation, Sufentanil, Off-pump coronary artery bypass grafting, Opioids consumption

## Abstract

**Background:**

High doses of long-acting opioids were used to facilitate off-pump coronary artery bypass grafting procedure, which may result in opioid-related adverse events after surgery. Transcutaneous electrical acupoint stimulation (TEAS) had been reported to be effective in reducing intraoperative opioids consumption during surgery. The aim of this study is to assess whether TEAS with difference acupoints can reduce the doses of opioid analgesics.

**Methods:**

This was a multicenter, randomized, controlled, double-blind trial. Patients underwent off-pump coronary artery bypass grafting under general anesthesia were enrolled. Eligible patients were randomly and equally grouped into sham acupuncture group (*n* = 105), regional acupoints combination group (*n* = 105), or distal–proximal acupoints combination group (*n* = 105) using a centralized computer-generated randomization system. Transcutaneous electrical acupoint stimulation was applied for 30 min before anesthesia induction. The primary outcome was the doses of sufentanil during anesthesia. Secondary outcomes included the highest postoperative vasoactive-inotropic scores within 24 h, intraoperative propofol consumption, length of mechanical ventilation, duration of cardiac care unit and postoperative hospital stay, incidence of postoperative complications, and mortality within 30 days after surgery.

**Results:**

Of the 315 randomized patients, 313 completed the trial. In the modified intention-to-treat analysis, the doses of sufentanil were 303.9 (10.8) μg in the distal–proximal acupoints group, significantly lower than the sham group, and the mean difference was − 34.9 (− 64.9 to − 4.9) μg, *p* = 0.023. The consumption of sufentanil was lower in distal–proximal group than regional group (303.9 *vs.* 339.5), and mean difference was − 35.5 (− 65.6 to − 5.5) μg, *p* = 0.020. The distal–proximal group showed 10% reduction in opioids consumption comparing to both regional and sham groups. Secondary outcomes were comparable among three groups.

**Conclusion:**

Transcutaneous electrical acupoint stimulation with distal–proximal acupoints combination, compared to regional acupoints combination and sham acupuncture, significantly reduced sufentanil consumption in patients who underwent off-pump coronary artery bypass grafting surgery.

**Supplementary Information:**

The online version contains supplementary material available at 10.1186/s13741-024-00427-2.

## Introduction

Off-pump coronary artery bypass grafting (CABG) is a minimally invasive procedure performed on a beating heart without cardiopulmonary bypass (CPB). Consequently, considerable hemodynamic fluctuations are common during the procedure (Go et al. 2013; Kurowicki et al. 2020). Traditionally, high doses of long-acting opioids were used to facilitate off-pump CABG procedure (Mathison et al. 2000). However, it may result in opioid-related adverse events after surgery (Shu et al. 2013), with a documented incidence of up to 31.8% following CABG surgery (Barisin et al. 2007). Complications of gastrointestinal, central nervous system, or respiratory systems, pruritus, and urinary retention are the most common adverse events. Accumulating evidence supported the efficacy of multimodal opioid consumption strategies in reducing opioid consumption in cardiac surgery patients (Wick et al. 2017). The multimodal opioid consumption approaches are now recommended by the recent enhanced recovery after surgery (ERAS) guidelines for perioperative care in cardiac surgery (Engelman et al. 2019).

Transcutaneous electrical acupoint stimulation (TEAS) had been reported to be effective in reducing intraoperative opioids consumption and alleviate postoperative opioid-associated adverse events after surgery (Devlin and Roberts 2009). Moreover, mounting evidence has verified the protective effects of electroacupuncture in cardiac surgery, including attenuated myocardial injury (Bovill et al. 1984), lower incidence of postoperative cognitive dysfunction (Grunkemeier et al. 2007), and reduced duration of mechanical ventilation as well as the length of ICU stay (Baum et al. 2021). Combination of acupoints stimulation enhanced the efficacy of acupuncture, as synergistic effects were observed (Wolters et al. 1982). In lumbago rats, it was demonstrated that stimulation of distal–proximal combination of acupoints was more potent in raising the pain threshold than stimulation of regional acupoints combination (Taylor et al. 2019; Burkhart et al. 2010).

The purpose of this study was to compare the effects of TEAS on distal–proximal acupoints on intraoperative opioids consumption in patients undergoing off-pump CABG to those of regional acupoints combination or sham TEAS in multicenter settings, in order to testify the generalization of TEAS on the distal–proximal acupoints combination strategy. Because acupuncture was proven to have many other benefits, therefore, the endpoints such as hemodynamics, cardiac function, incidence of postoperative complications, and mortality were also included in this clinical trial.

## Methods

### Study design

We conducted an investigator-initiated, multicenter (five sites), randomized, controlled 3-arm trial in China. This study was approved by the Ethics Committee of The Xijing Hospital (KY20140420-X-1) and was registered with ClinicalTrials.gov (NCT02443220, date of registration: May 13, 2015). The study was registered prior to the first patient enrollment. Written informed consents were obtained from all participants before any experimental procedures. The first author attests to the accuracy and completeness of the data and for the fidelity and adherence of the trial to the protocol. The study is reported according to the Consolidated Standards of Reporting Trials (CONSORT) and the Standards for Reporting Intervention of Clinical Trials of Acupuncture (STRICTA) reporting guidelines.

### Participants

Eligible study participants were patients with angiographically identified coronary artery disease scheduled for elective off-pump coronary artery bypass grafting surgery. Inclusion criteria included an American Society of Anesthesiologists score of less than IV and scheduled for first-time open-heart surgery. Exclusion criteria included urgent or emergent surgery; life expectancy < 1 year at the time of enrollment; hemodynamic instability, defined as systolic blood pressure < 90 mm Hg; supported with intra-aortic balloon pumping or ventricular assisted device; severe hepatic or renal dysfunction; severe systemic infection; contraindicated for TEAS, such as wound or infection at the selected acupoints; and suffering from nervous system diseases or with abnormal mental state.

### Randomization and blinding

Eligible patients were randomly assigned with a 1:1:1 ratio to receive TEAS on distal–proximal acupoints, TEAS on regional acupoints or sham acupuncture, respectively. Randomization was performed through a secure randomization system with allocation concealment, using permuted block randomization, and stratified by accrual site.

The participants, outcome assessors, data managers, statisticians, and study monitors were blinded to the allocation. The clinical research coordinator (CRC) and acupuncture therapists were not blinded to group allocation. Study site investigators who were blinded to group allocation enrolled patient into study and collected clinical outcome data. The acupuncture therapist was the only unblinded investigator who opened the sealed envelope right before the anesthesia induction once the patient has consented to participate and then assigned the treatment group accordingly. The transcutaneous electrical acupoint stimulation (TEAS) intervention was administered by this acupuncture therapists in the pre-anesthesia preparation room. To ensure blinding during stimulation, the electronic acupuncture treatment instrument was securely encased in a box, concealing the stimulation current. Blinding was maintained until completion of analysis.

### Interventions

Participants were recruited at the time of the decision for elective off-pump CABG surgery, and randomization occurred prior to the commencement of the surgery, after the patients entered into the pre-anesthesia preparation room. This maintained the allocation concealment to great extent. After randomization, the TEAS intervention was delivered by acupuncture therapists with at least 2 years of clinical experience.

The TEAS protocol was developed using a Delphi method. The treatment strategy (acupoints selection and frequency of electrical stimulation) was based on traditional Chinese medicine and consensus of clinical experts, and details are shown in Fig. S1 in Supplement. Participants in the distal–proximal acupoints combination group received acupuncture at Danzhong (CV17, located on the sternum) (Kurono et al. 2011; Li et al. 2021) and bilateral Hegu (LI4, located between the base of thumb and index fingers) (Guangjun et al. 2012, 2014). This combination of acupoints is believe to promote cardiac vagal function and enhance analgesia effects when stimulated. Participants in the regional acupoints combination group received acupuncture at Danzhong (CV17) and Juque (CV14, located at 6 cun above umbilicus) (Zou et al. 2018). This combination of acupoints is believe to reduce the heart rate and alleviate chest pain when stimulated.

After skin sterilization, conductive electrodes pads were pasted on the selected acupoints. The local acupoints were electrically stimulated with Hwato Electronic Acupuncture Treatment Instrument (model no. SDZ-V, Suzhou Medical Appliance Co., Ltd., Suzhou, China). The frequency appeared alternately at sparse-dense wave (2/15 Hz), and the intensity gradually increased from 1 mA until the maximum current that the patient could tolerate. The maximum current that individual patients can tolerate was recorded. Appropriate stimulation on acupoints would induce a sensation of *de qi*, a composite local sensation of soreness, numbness, distension, heaviness, and other sensations. This is believed to be the essential component for the efficacy of acupuncture.

Patients in the sham group were administered sham acupuncture with electrodes pads pasted at CV17 and bilateral LI4 and connected to the electronic acupuncture treatment instrument, but without stimulation. TEAS sessions lasted 30 min prior to anesthesia induction. To blind the patients as much as possible, only patients who had never been exposed to TEAS were enrolled, and they were informed before acupuncture treatment that they may be sensitive or insensitive to TEAS. During the 30-min treatment process, the patients in sham group were asked if the stimulation was tolerable for three times to maximize the blinding. During stimulation, the electronic acupuncture treatment instrument is encased in a box; therefore, the stimulation current was covered and concealed. Stimulation-related adverse events were monitored and documented by the observation of researchers and the self-report of the patients.

Bolus injection of 0.03 mg/kg midazolam, target-controlled infusion (TCI) of propofol with plasma concentration of 1.0–2.0 ug/ml (Marsh model (Guggenberger et al. 2006)), and sufentanil with initial effect site concentration of 0.2 ng/ml (Gepts model (Kumar et al. 2017)) and increased 0.2 ng/ml per minute according to the change of bi-spectral index (BIS) were used for anesthesia induction. When patients lost consciousness, 0.6 mg/kg rocuronium was administered to facilitate endotracheal intubation. During operation, the BIS was maintained at 40 to 60. The effect site concentration of sufentanil was adjusted based on hemodynamics, BIS, and anesthesiologist experience, but propofol plasma concentration was kept steady.

Intraoperative hemodynamic parameters, including demographic characteristics, preoperative comorbidities, heart rate (HR), mean arterial pressure (MAP), BIS during the anesthesia, time of anesthesia and operation, and grafted numbers of coronary, were collected to describe diversity of the study population and the comparability of different intervention arms. HR, MAP, and BIS were recorded respectively at T0 (right before induction), T1 (loss of consciousness, LOS), T2 (right before intubation), T3 (3 min after intubation), T4 (incision), T5 (1 min after median sternotomy), T6 (after dissection of internal mammary artery), T7 (right before partial clamping of aorta), T8 (clamp releasing), T9 (5 min after reperfusion), T10 (closure of sternum), and T11 (ends of surgery).

### Outcomes

The primary endpoint was the intraoperative total sufentanil consumption. Secondary endpoints were the highest vasoactive-inotropic scores (VIS) at 24 h after surgery, intraoperative propofol consumption, length of mechanical ventilation, length of CCU and postoperative hospital stay, incidence of serious complications within 30 days after surgery, and mortality at 30 days. The VIS was calculated based on the doses of dopamine, dobutamine, epinephrine, norepinephrine, vasopressin, and milrinone (Table S1 in Supplement). Serious complications included myocardial infarction, new onset of cardiac dysfunction (ejection fractions less than 35%, heart failure, or pericardial tamponade), delirium, shock, moderate-to-severe acute kidney injury (Kidney Disease: Improving Global Outcomes stages 2 or 3), liver dysfunction (liver transaminase increased more than twice as much as preoperative baseline), pulmonary complications (pulmonary infection, atelectasis, or pleural effusion), infection, or sepsis.

### Statistical analysis

The main hypothesis of this study was that stimulation of distal–proximal acupoints would lead to a reduction in intraoperative sufentanil consumption compared to regional acupoints stimulation and sham acupuncture. To test this hypothesis, two comparisons were made between the three intervention groups, and an alpha of 0.025 (two-sided) was used for the Bonferroni correction in the sample size calculation. A group difference of 45.4 μg of sufentanil consumption, corresponding to a 15% reduction, was predefined based on the results of a pilot study. To detect or reject this predetermined group difference with 80% power (two-sided tests; alpha = 0.025), 315 participants (105 per group) were required. Since the probability of missing or loss to follow-up in the primary endpoint was negligible, the sample size was not expanded. The sample size was performed using Stata version 15.1.

The descriptive statistics were summarized in the following manner where continuous variables were reported as means (standard deviations) or medians (inter-quartile ranges) where appropriate. Categorical variables were presented as frequencies (percentages). Normality was established via Shapiro–Wilk tests or Fisher exact tests accordingly.

The primary analysis was carried over the intention-to-treat population. Comparisons of the distal–proximal group were carried out against both the regional and sham groups. The mean differences were reported with 95% confidence intervals and *p*-values. The sufentanil consumption was demonstrated to be skewed to the left in the pilot study; the primary endpoint was thus further analyzed via the generalized linear model (GLM) over Gaussian family with log link. Covariates including weights, anesthesia duration, and study sites were adjusted for as potential confounding effects. Estimates of relative risk and marginal means were reported adjusting for weights, anesthesia duration, and study sites.

The secondary outcomes were compared in the similar manner; the distal–proximal group was compared against both the regional and sham groups respectively. The standardized mean differences were presented with 95% confidence intervals. The trends of the hemodynamic measurements were shown using smoothed lines via spline curves. Further explorations of behavior of hemodynamic measurements were done via log-Gaussian generalized linear models adjusting for the baseline information, group indicator, time points, and group-time interactions. The ICU and postoperative hospital stays were evaluated via Cox regression taking account of group indicator (distal–proximal group as reference category) and central effects (referenced on center 1).

The robustness of the study results was governed by the following sensitivity analyses. The primary endpoint was conducted in the per-protocol population including only those participants who completed the treatment originally allocated. A generalized linear model with additional center effect adjusted for was also used to re-estimate the effects of intervention removing the impacts clustered within each center.

The post hoc subgroup analyses further investigated how different groups of patients react to the intervention assigned, e.g., how different gender is affected. The following subgroups were considered: sex, age (< 65 years or ≥ 65 years), participating sites, levels of NYHA, clamping aorta or not, and European System for Cardiac Operative Risk Evaluation (EuroSCORE I, < 4 or ≥ 4). No primary outcomes were missing. Missingness in hemodynamic parameters were imputed using the last observation carried forward method. Multiple imputation was used for other missing data.

All statistical analyses were performed at 5% significance level using IBM SPSS statistics 26 or R version 4.1.0 (www.r-project.org). For the primary endpoint, Bonferroni approach was adopted for multiple comparisons (alpha = 0.025) for each independent comparison of distal–proximal acupoints stimulation vs sham acupuncture and distal–proximal acupoints stimulation vs regional acupoints stimulation. Analyses for secondary or post hoc analyses were not adjusted; therefore, they were interpreted as exploratory.

## Results

**Fig. 1:**
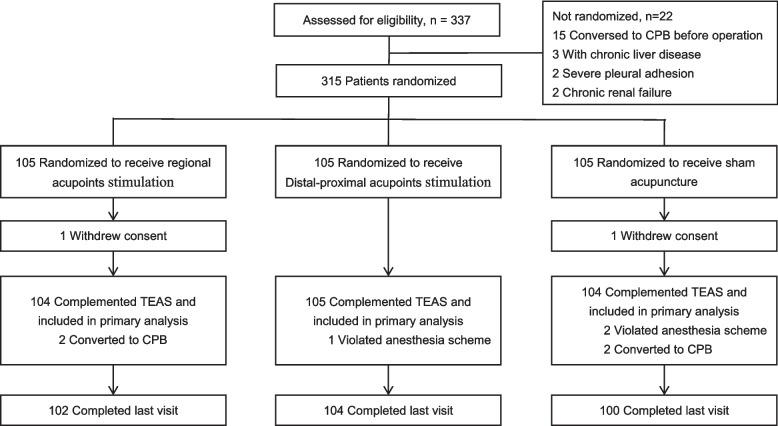
Flowchart showing trial group assignments, loss of follow-up, treatment completion, and protocol deviation. Eligible for randomized, 313 patients were included in modified intention-to-treat analysis, whereas 306 patients were included in the per protocol analysis

A total of 337 candidates were screened for eligibility from October 2017 to September 2018. Of them, 22 were excluded due to ineligibility for various reasons (details see Fig. 1), leaving 315 patients eligible for randomized (105 in each group, Fig. 1). The number of dropouts or protocol violation at each stage and the number assessed for primary end point are presented in Fig. 1. Finally, 313 patients were included in modified intention-to-treat analysis, whereas 306 patients were included in the per protocol analysis. There was no substantial difference in the dropout rates among the groups. The last follow-up visit was completed on August 15, 2019. All subjects completed outcome measurements at 30 days.

Patient baseline characteristics are demonstrated in Table 1 and were balanced among groups. There were 70 women (22.2%) and 245 men (77.8%) with a mean age of 61.7 (8.6) years included in the study. The delivery of treatment and compliance with the intervention were evenly distributed among groups.

**Table 1:** Baseline characteristics of randomized population

Baseline characteristics		Regional group *n* = 105	Distal–proximal group *n* = 105	Sham group *n* = 105
Age (years)		64.0 (57.5, 68.0)	63.0 (55.0, 67.0)	61.0 (57.0–67.0)
Female, *n* (%)		22 (21.0)	23 (21.9)	25 (23.8)
BMI (kg/m^2)^		25.8 (23.3, 28.2)	25.5 (23.6, 27.4)	25.3 (23.3, 27.4)
NYHA, *n* (%)				
II		62 (59.0)	56 (53.3)	59 (46.7)
III		43 (41.0)	49 (46.7)	45 (43.3)
EuroSCORE I		4.0 (2.0, 5.0)	4.0 (3.0, 5.0)	4.0 (2.75, 5.0)
Previous MI, *n* (%)		34 (33.0)	34 (32.4)	23 (22.1)
Uncorrected valvular heart disease, *n* (%)		30 (28.6)	34 (32.4)	34 (32.4)
Previous stock, *n* (%)		23 (21.9)	20 (19.0)	22 (21.0)
Diabetes, *n* (%)		38 (36.2)	41 (39.0)	44 (41.9)
Hypertension, *n* (%)		77 (73.3)	67 (63.8)	73 (69.5)
Dyslipidemia, *n* (%)		14 (13.3)	25 (23.8)	17 (16.2)
Smoking, *n* (%)	Never	38 (36.2)	34 (32.3)	36 (34.3)
	Within 12 m	46 (43.8)	51 (48.6)	43 (41.0)
	More than 12 m	19 (18.1)	20 (19.0)	24 (22.9)
Previous cardiac surgery, *n* (%)		6 (5.7)	1 (1.0)	6 (5.7)
Abnormal ECG, *n* (%)		85 (81.0)	86 (81.9)	91 (86.7)
Intraoperative characteristics				
Length of operation (minutes)		202.0 (148.0, 260.0)	210.0 (145.0, 290.0)	210.0 (150.0, 275.5)
Length of anesthesia (minutes)		252.0 (212.7, 308.1)	256.0 (189.0, 298.8)	233.0 (174.3, 283.6)
Grafted numbers of coronary		3.0 (2.0, 3.0)	3.0 (2.0, 4.0)	3.0 (2.0, 3.0)

Values are median (IQR), unless stated otherwise. Abbreviations: *IQR*, interquartile range; *BMI*, body mass index; *MI*, myocardial infraction; *NYHA*, New York Heart Association classification; *EuroSCORE*, European System for Cardiac Operation Risk Evaluation; *LVEF*, left ventricle ejection fraction

### Primary outcome

**Fig. 2:**
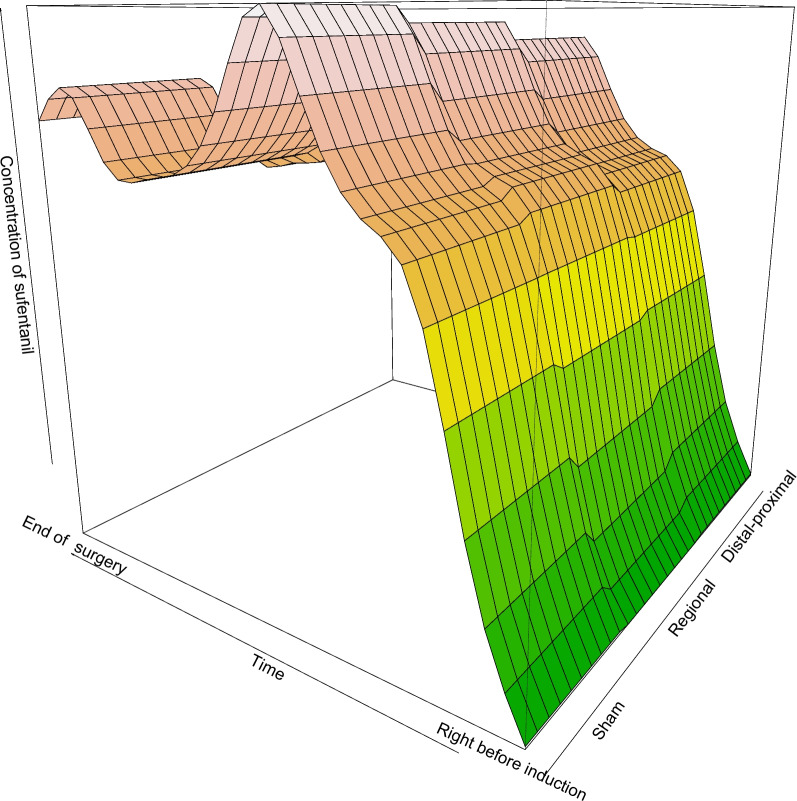
Surface plot of concentration sufentanil vs time group. The contour plot shows the relationship between the time, group, and the concentration of sufentanil. The concentration of sufentanil during anesthesia was lower in distal–proximal group

The primary end point was analyzed in the modified intention-to-treat set and per protocol population (Table 2). In the modified intention-to-treat set, the total sufentanil consumption in distal–proximal, regional, and sham groups were 303.94 (10.80), 339.49 (10.87), and 338.84 (10.87) μg, respectively (Table S2 in Supplement). The total sufentanil consumption was reduced in distal–proximal acupoints stimulation group as compared with regional acupoints stimulation group (mean difference: − 35.54 μg; 95% *CI*, − 65.56 to − 5.53 μg; *p* < 0.025) and sham group (mean difference: − 34.89 μg; 95% *CI*, − 64.93 to − 4.85 μg; *p* < 0.025) (Table 2 and Table S3 in Supplement). This pattern of findings for the primary outcome was unchanged in the per-protocol population (Table 2 and Table S3 in Supplement). The generalized linear model with log-Gaussian family also guaranteed such effect; upon adjustment of weight and anesthesia times, the effect distal–proximal group still shows the significant lowest effect on sufentanil consumption (Figure S2 in Supplement). The estimates are robust as they are consistent on adjustment of central effects (Figure S3 in Supplement). The contour plot of Fig. 2 shows the relationship between the time, group, and the concentration of sufentanil. The concentration of sufentanil during anesthesia was lower in distal–proximal group.

**Table 2:** Primary outcome measurements of modified intention to treat set and per protocol set

Group	Regional group	Distal–proximal group	Sham group	Distal–proximal *vs* regional		Distal–proximal *vs* sham	
MITT, *n* = 313	*n* = 104	*n* = 105	*n* = 104	Mean difference (95% CI)	p*	Mean difference (95% CI)	p*
Dose of sufentanil (μg)	339.5 (10.9)	303.9 (10.8)	338.8 (10.9)	− 35.5 (− 65.6, − 5.5)	0.020	− 34.9 (− 64.9, − 4.9)	0.023
PP, *n* = 306	*n* = 102	*n* = 104	*n* = 100				
Dose of sufentanil (μg)	338.6 (11.0)	303.6 (10.9)	339.4 (11.1)	− 35.0 (− 65.4, − 4.6)	0.024	− 35.9 (− 66.4, − 5.3)	0.021

*Abbreviations: SE* standard error, *MITT* modified intention-to-treat analysis, *PP* per protocol

Values are marginal mean (SE)

^*^Multiple pairwise comparisons were adjusted by Bonferroni approach with *p* < 0.025 considered as significant

### Secondary and exploratory outcomes

For the secondary outcomes, no significant differences were seen among three groups (Table 3). The intraoperative propofol consumption was comparable among the patients in three groups. Length of mechanical ventilation, duration of cardiac care unit, and postoperative hospital stays were not significantly different across the three groups. Pulmonary complications, infection, liver dysfunction, and acute kidney injury were the most common postoperative complications, with incidences ranging from 10 to 20%. Specifically, the incidences of all complications occurred statistically equivalently across three groups. One patient in regional and distal–proximal group (0.98% and 0.96%, respectively) and three patients (2.88%) in sham group died within 30 days after surgery. No significance in mortality differences was found. Despite no significant interaction was found between intervention and time for MAP, HR, and BIS and concentration of sufentanil (Figs. S3 and S4 in Supplement), the tendency of differences can still be seen in MAP, HR, and concentration of sufentanil.

**Table 3:** Second outcomes were analyzed in modified intention-to-treat analysis

Group	Regional group *n* = 104	Distal–proximal group *n* = 105	Sham group *n* = 104	Distal–proximal *vs* regional	Distal–proximal *vs* sham
				SMD (95% *CI*)	SMD (95% *CI*)
Postoperative VIS at 24 h	3.5 (2.0, 4.9)	4.5 (2.0, 5.0)	4.5 (2.7, 5.9)	0.22 (− 0.05, 0.50)	0.13 (− 0.14, 0,40)
Dosage of propofol (mg)	561.0 (359.5, 847.5)	581.5 (383.5, 923.5)	470.5 (274.8, 727.3)	0.00 (− 0.27, 0.27)	0.22 (− 0.06, 0.49)
Length of mechanical ventilation (h)	16.3 (8.8, 19.0)	15.8 (7.8, 19.1)	15.9 (10.8, 19.9)	0.12 (− 0.15, 0.39)	0.02 (− 0.25, 0.29)
Length of CCU stays (h)	40.0 (22.8, 46.0)	41.0 (27.8, 45.1)	41.0 (25.3, 67.8)	0.05 (− 0.23, 0.32)	0.19 (− 0.08, 0.46)
Length of postoperative hospital stays (day)	9.0 (7.0, 11.0)	9.0 (7.0, 12.0)	9.0 (8.0, 11.8)	0.17 (− 0.10, 0.45)	0.02 (− 0.26, 0.29)
Postoperative complications, *n* (%)					
Myocardial infarction	19 (18.3)	27 (25.7)	19 (18.3)	0.18 (− 0.09, 0.46)	0.16 (− 0.12, 0.43)
Cardiac dysfunction	2 (1.9)	4 (3.8)	5 (4.8)	0.16 (− 0.11, 0.43)	0.00 (− 0.27, 0.28)
Delirium	0 (0.0)	1 (1.0)	3 (2.9)	0.14 (− 0.13, 0.41)	0.15 (− 0.13, 0.42)
Stroke	0 (0.0)	2 (1.9)	1 (1.0)	0.20 (− 0.08, 0.47)	0.08 (− 0.20, 0.35)
Acute kidney injury	10 (9.6)	12 (11.4)	10 (9.6)	0.06 (− 0.22, 0.34)	0.06 (− 0.21, 0.34)
Liver dysfunction	10 (9.6)	19 (18.1)	15 (14.4)	0.25 (− 0.02, 0.53)	0.10 (− 0.17, 0.38)
Pulmonary complication	19 (18.3)	21 (19.0)	13 (12.5)	0.04 (− 0.24, 0.31)	0.20 (− 0.08, 0.48)
Infection	20 (19.2)	21 (19.0)	18 (17.3)	0.02 (− 0.26, 0.29)	0.06 (− 0.22, 0.33)
Other complication	2 (1.9)	3 (2.9)	0 (0.0)	0.14 (− 0.14, 0.41)	0.06 (− 0.22, 0.33)
Mortality within 30 days, *n* (%)	1 (1.0)	1 (1.0)	3 (2.9)	0.00 (− 0.27, 0.28)	0.09 (− 0.19, 0.36)

*Abbreviations: SMD* standard mean difference, calculating as difference in mean outcome between groups divided by standard deviation of outcome among participants, *VIS* vasoactive-inotropic scores, *CCU* cardiac care unit

Values are median (IQR), unless stated otherwise

### Subgroups

**Fig. 3:**
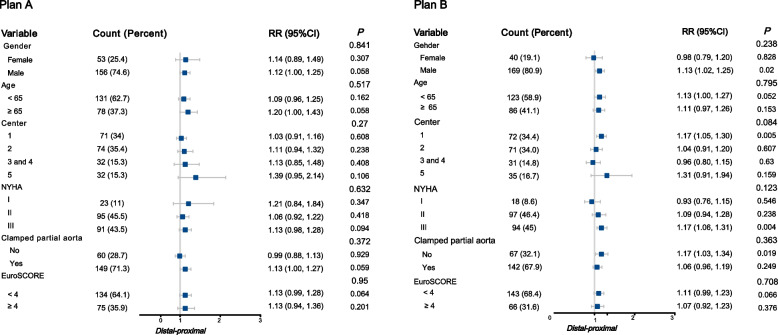
Subgroup analysis of total doses of sufentanil with GLM in modified intention-to-treat population. None of the subgroups showed significant interactions. Plan A, subgroup analysis of total doses of sufentanil with GLM between distal–proximal group and regional group. Plan B, subgroup analysis of total doses of sufentanil with GLM between distal–proximal group and sham group. The sufentanil consumption effect of distal–proximal group was more prominent when compared with regional and sham groups in male and patients whose NYHA classification was III and underwent surgery in center 1

The interactions in relation to the treatment effects of distal–proximal acupoints stimulation vs regional acupoints stimulation and distal–proximal acupoints stimulation vs sham stimulation on consumption of sufentanil were investigated in post hoc subgroups. None of the subgroups showed significant interactions. However, some subgroup effects were shown significant. For example, the sufentanil consumption effect of distal–proximal group was more prominent when compared with regional and sham groups in male and patients whose NYHA classification was III and underwent surgery in center 1 (Fig. 3, Tables S4 and S5 in Supplement). No other subgroup effects were found significant in the specified stratum, including age, aorta clamping, or EuroScore I.

### Adverse events and quality of blinding

Stimulation-related adverse events include continuous post-electrostimulation sensation and skin numbness, allergic reaction, redness, swelling, pain, or other injury on the skin during acupuncture treatment. There were no stimulation-related adverse events or other equipment malfunction reported in any of the three groups during the treatment. No serious adverse events were identified that was TEAS related.

## Discussion

In this multicenter randomized controlled trial, we investigated the effect of TEAS on opioid consumption in patients scheduled for off-pump CABG surgery under total intravenous anesthesia. Comparing with regional acupoints combination and sham acupuncture, TEAS with distal–proximal acupoints combination significantly reduced total sufentanil consumption. The study testified the opioid consumption effect of TEAS with different acupoints combination in patients undergoing off-pump CABG surgery in a multicenter setting. The study provides evidences for the acupoints selection on how the TEAS or acupuncture drug balanced anesthesia should be applied to clinical practice.

TEAS with distal–proximal acupoints combination was shown to effectively reduce the dose of opioids as compared with regional acupoints combination and sham acupuncture. These findings indicate that the opioid-sparing effect of TEAS is associated with selection of acupoints. As previously established, acupuncture has been increasingly used to assist anesthesia during surgeries, thanks to its potent analgesic effects (Wang et al. 2014; Zhou et al. 2011; Zhang et al. 2014).

Different acupoints combination determines the clinical efficacy of acupuncture (Xuefen et al. 2020; Du et al. 2022). For example, the regional acupoints combination of ZhongFu (LU1), LieQue (LU7), and XiMen (PC4) acupoints for electroacupuncture started 15–20 min prior to surgical incision to the end of cardiopulmonary bypass-assisted cardiac surgery and only consumed 13% of the fentanyl required in the control group without electroacupuncture (Zhou et al. 2011). Similarly, the opioids consumption in the distal–proximal group in our study was shown to have reduced by approximately 10% comparing to the sham group. Empirical evidence of traditional Chinese medicine and clinical experiences both suggested that stimulation on distal–proximal acupoints combination (acupoints distal and adjacent to lesion position) has synergistic effects and is more potent than regional acupoints combination (Xuefen et al. 2020; Du et al. 2022; Wu et al. 2020). In a randomized controlled trial, TEAS with distal–proximal acupoints combination of Hegu (LI4), Neiguan (PC6), and Zusanli (ST36) for 30 min before anesthesia reduced remifentanil requirement for 39% in patients undergoing sinusotomy as compared with no TEAS (Wang et al. 2014). The reason behind this could be as suggested by accumulating evidences on the analgesia effect of acupuncture; it is mediated by reducing the excitability of sympathetic nervous, inhibiting the production of catecholamine, and increasing the levels of adrenocorticotropic hormone, cortisol, endothelin, and adenosine (Visvardis et al. 2004; Risch et al. 2001). Our study further contributes to the experience by showing a significant opioids consumption reduction in the distal–proximal group comparing to the regional group by approximately 10%.

The study has not found significant results in the secondary outcomes. Although previous studies have reported that the incidence of postoperative pulmonary complications, length of ICU stay, and the overall medical costs were reduced (Zhang et al. 2014), this should not establish a major violation as the sample size calculated based on the primary outcome does not provide sufficient power for solid evidence in these secondary outcomes. Tendencies of reduction comparing to sham group were also shown in length of CCU stays in our study. Moreover, length of mechanical ventilation, length of postoperative hospital stays, cardiac dysfunction, delirium, and other complications all showed reduction tendencies comparing to the sham group. Further investigations shall be conducted to draw firm evidences in these aspects.

The study has also shown tendency that different subgroups benefit differently. For example, males, over 65 years old group, the higher NYHA group, and aorta partial clamped group tend to benefit more. This may be explained as these groups are commonly administrated less opioid dosages as the body functions are sensitive to drugs prescribed and treatment given. The temporal trends in hemodynamic suggested possible diversions among groups in different time points, but firm conclusions still request designs with larger sample sizes and more precise records.

Up until now, very few studies have compared the effects of distal–proximal acupoints combination to any other acupoints combination strategies, to the best of our knowledge. Therefore, our study fills the gap of evidence-based support to the benefit of TEAS on distal–proximal acupoints combination to the regional acupoints combination via a sophisticated RCT in multiple centers. The blinding is also carefully concerned and conducted with the best effort. For example, all patients were asked if the stimulation was tolerable for several times during TEAS treatment process. However, there are several limitations in our study. First, the use of electrical stimulation may make it difficult to separate activation of acupoints from non-acupoints since the electrical current spreads between acupoints. This means that the anesthetic-sparing effects of TEAS may be blunt as they rely highly on the accuracy of locations of acupoints. Second, the length of the TEAS intervention was short. This may be the potential explanation for failure to detect significant statistical differences as refer to the postoperative complications or mortality among three groups in our study. Third, although the patients were blinded to the group allocation in this study, the blinding assessment was not conducted when the intervention finished. We do not take this as a major disadvantage to our study due to methods taken during procedures (e.g., asking patients about current described above), and the primary outcome being the intraoperative opioid consumption limits the subjective bias injected from the patients. Also, the ultimate goal of this research is to find the optimal acupoints combination in patients undergoing off-pump CABG, and the previous study findings suggested that the sufentanil-sparing effect in distal–proximal acupoints combination group was significantly better than both the regional and sham group. No comparison was made between the regional acupoints combination group and sham group. Fourth, our initial estimate was a 15% reduction in the dosage of sufentanil when using a combination of distal–proximal acupoints. However, the actual reduction achieved was only 10%. While a 10% reduction in opioid dosage may demonstrate statistical significance, determining its clinical significance would require careful consideration within the specific clinical context. Additionally, our investigation did not identify any evidence of opioid-related side effects such as postoperative nausea and vomiting, respiratory depression, or pruritus. It is important to note that our study included patients undergoing off-pump coronary artery bypass grafting (CABG), who were subsequently transferred to the coronary care unit (CCU) for further evaluation and treatment. Therefore, complete information on all short-term opioid side effects is not available.

## Conclusion

In this multicenter, randomized controlled trial, significant reduction of intraoperative opioids consumption was observed in TEAS with distal–proximal acupoints combination compared to regional acupoints combination in off-pump CABG surgery. Potential benefit populations are pointed out, but future studies are needed for firm conclusions. The future study should also focus on the effects of TEAS with distal–proximal acupoints combination on long-term outcome of patients undergoing cardiac surgery.

### Supplementary Information

Additional file 1: Figure S1. Location of the Acupoints. Figure S2. Estimate total dose of sufentanil between three groups. Figure S3. Estimate total dose of sunfentanil between three groups. Figure S4. MAP, HR, BIS and Concentration of sufentanil during anesthesia. Figure S5. Interaction between intervention and time for MAP, HR, BIS and Concentration of sufentanil. Table S1. The version of vasoactive-inotropic score (VIS). Table S2. Marginal Means of total sufentanil consumption. Table S3. Difference(95% CI) of total sufentanil consumption among three groups. Table S4. Subgroup analysis of total sufentanil consumption with GLM between distal-proximal group and regional group. Table S5. Subgroup analysis of total sufentanil consumption with GLM between distal-proximal group and sham group.

## Data Availability

No datasets were generated or analysed during the current study.
