# Supplementary material for: The use of transcutaneous electrical acupoint stimulation to reduce opioid consumption in patients undergoing off-pump CABG: a randomized controlled trial
Source: Perioper Med (Lond). 2024 Jul 5;13:68. doi: 10.1186/s13741-024-00427-2 (PMC11225385; doi:10.1186/s13741-024-00427-2)
Supplement: Supplementary file 1 — Additional file 1: Figure S1. Location of the Acupoints. Figure S2. Estimate total dose of sufentanil between three groups. Figure S3. Estimate total dose of sunfentanil between three groups. Figure S4. MAP, HR, BIS and Concentration of sufentanil during anesthesia. Figure S5. Interaction between intervention and time for MAP, HR, BIS and Concentration of sufentanil. Table S1. The version of vasoactive-inotropic score (VIS). Table S2. Marginal Means of total sufentanil consumption. Table S3. Difference(95% CI) of total sufentanil consumption among three groups. Table S4. Subgroup analysis of total sufentanil consumption with GLM between distal-proximal group and regional group. Table S5. Subgroup analysis of total sufentanil consumption with GLM between distal-proximal group and sham group. [file 13741_2024_427_MOESM1_ESM.docx]

**Online Supplementary Material**

**The use of Transcutaneous Electroacupuncture to reduce opioid consumption in Patients undergoing off-pump CABG**

**A Randomized Controlled Trial**

This supplementary material has been provided by the authors to give readers additional information about our work.

**Figure S1 Location of the Acupoints**


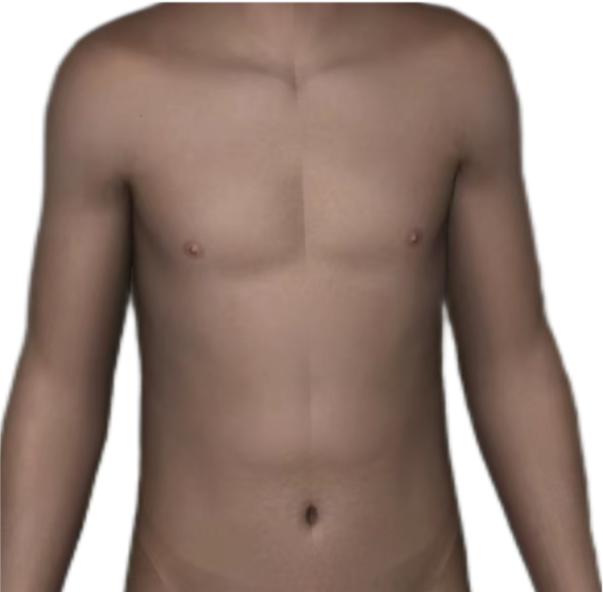


**CV 17**

0

8

6

**CV 14**


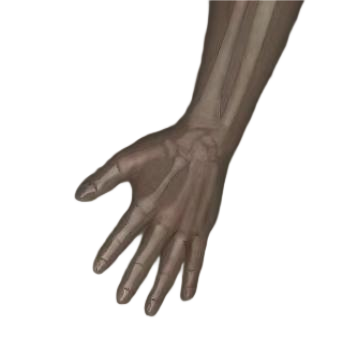


**LI 4**

second metacarpal bone

**CV 17:** It is located in the midline of the front of the body and the midpoint of the line between the two nipples. It’s commonly used for the treatment of anxiety or psychological strain-related somatic symptoms, such as palpitation, dyspnea and chest pain, suggesting an effect restoring or enhancing cardiac vagal function.

**CV 14:** It is located on the anterior midline of the upper abdomen, the top 1/4 of the belly button and CV17 connection. It is useful where somatic changes occur including pectoral pain, headaches, epigastric pain and nausea.

**LI 4:**  It is located at the mouth of the tiger on the back of the hand, trapped between the first metacarpal bone and the second metacarpal bone. It is important acupoints for analgesia, increasing the body pain threshold, alleviating microvascular spasms, and influencing the frontal cortex to participate in the discharge of the pain unit in the ventrolateral thalamic nucleus.

**Figure S2 Estimate total dose of sufentanil between three groups**

**
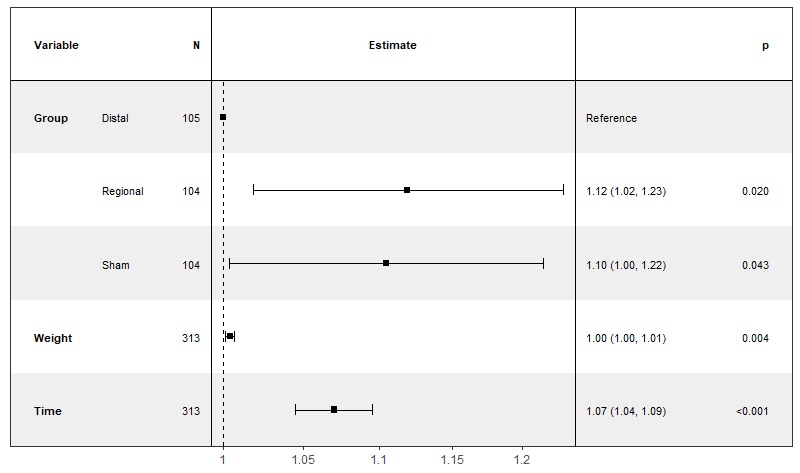
**

The generalized linear model with log-Gaussian family guaranteed the doses of sufentanil upon adjustment of weight and anesthesia times.**Figure S3 Estimate total dose of sunfentanil between three groups**

**
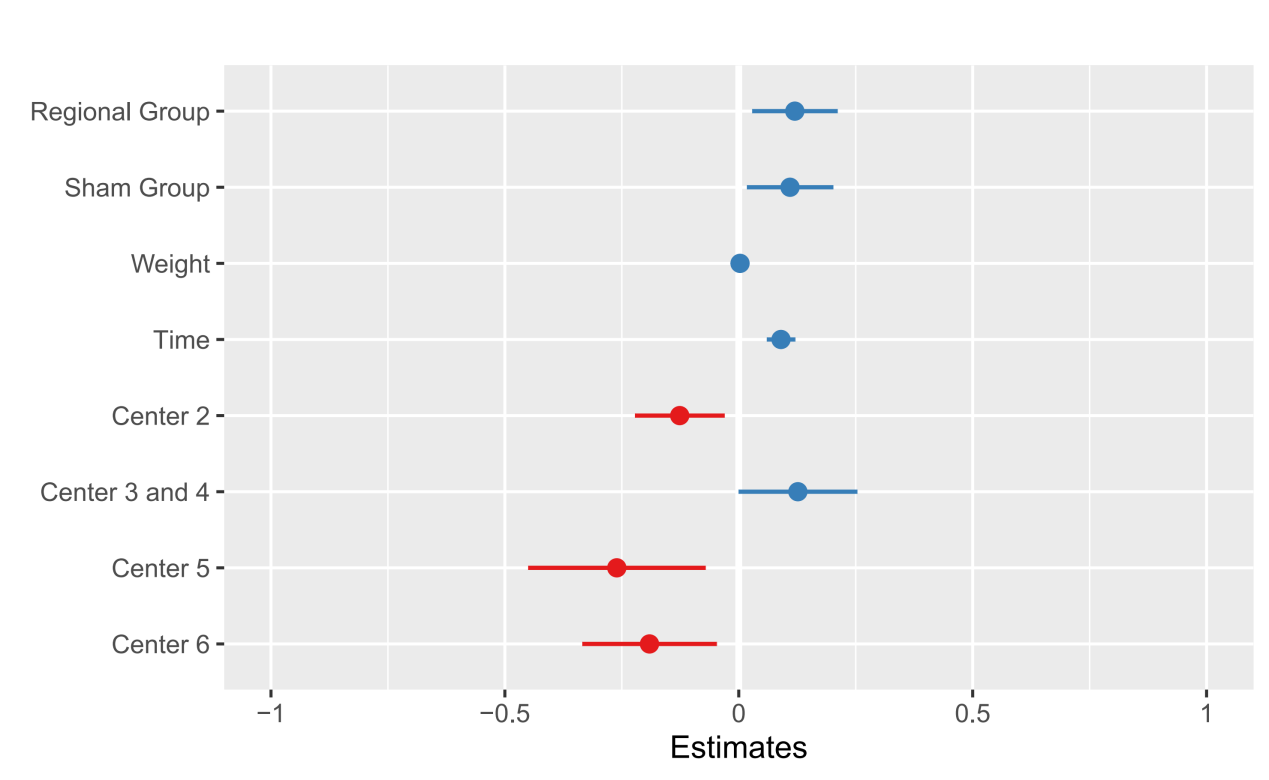
**

The generalized linear model with log-Gaussian family guaranteed the doses of sufentanil upon adjustment of weight, anesthesia times and central effects.

**Figure S4 MAP, HR, BIS and Concentration of sufentanil during anesthesia**


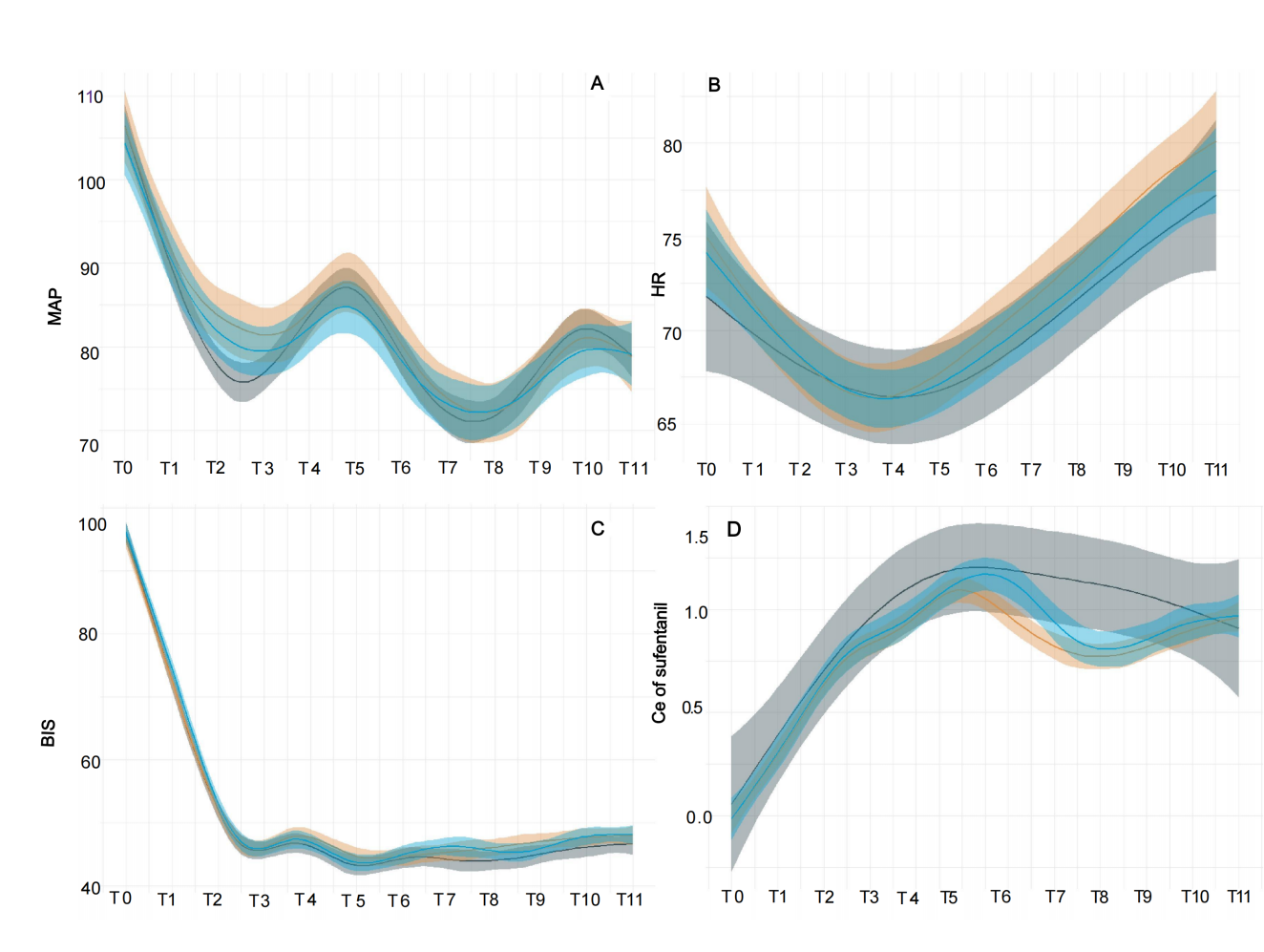


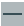
 Regional group
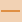
 Distal-proximal group
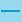
 Sham group

Plan A, MAP, mean arterial pressure; Plan B, HR, heart rate; Plan C, BIS, Bispectral index; Plan D, Ce, Concentration of sufentanil.

T0, right before induction; T1, loss of consciousness, LOS; T2, right before intubation; T3, 3min after intubation; T4, incision; T5, 1min after median sternotomy; T6, after dissection of internal mammary artery; T7, right before partial clamping of aorta; T8, clamp releasing; T9, 5 min after reperfusion; T10, closure of sternum; T11, ends of surgery.

**Figure S5 Interaction between intervention and time for MAP, HR, BIS and Concentration of sufentanil**


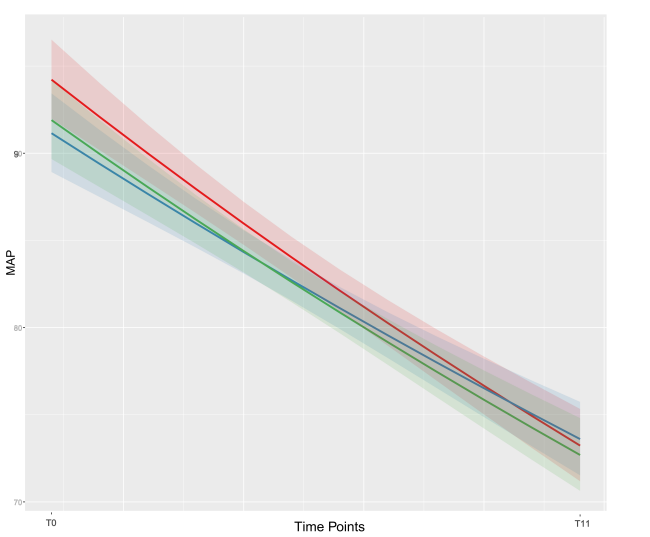

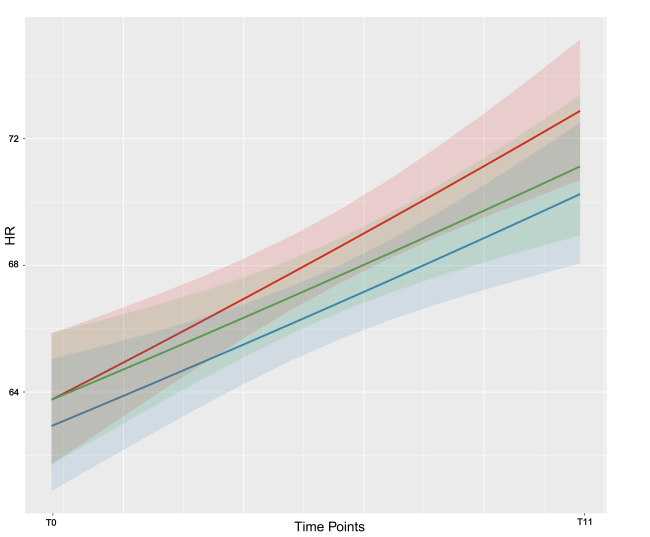

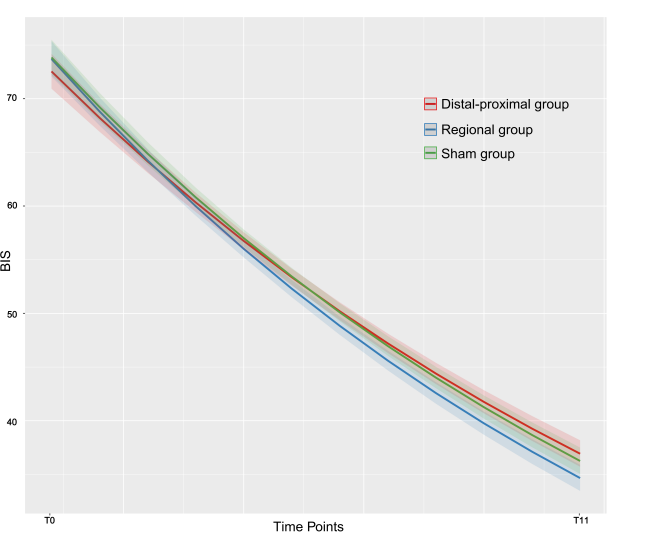

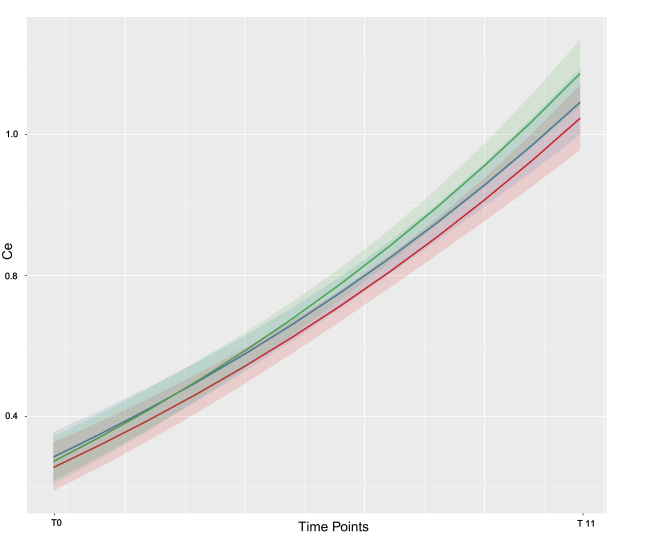


MAP, mean arterial pressure; HR, heart rate; BIS, bispectral index; Ce, concentration of sufentanil.

**Table S1 The version of vasoactive-inotropic score (VIS)**

| **Drug** | **Unit administrated** | Multiplier |
| --- | --- | --- |
| Dopamine | μg/ kg/ min | 1 |
| Dobutamine | μg/ kg/ min | 1 |
| Epinephrine | μg/ kg/ min | 100 |
| Norepinephrine | μg/ kg/ min | 100 |
| Vasopressin | U / kg/ min | 10,000 |
| Milrinone | μg/ kg/ min | 10 |

**Table S2 Marginal Means of total sufentanil consumption**

| Group | Marginal Means | Standard Error | 95% Confidence Interval | |
| --- | --- | --- | --- | --- |
|  |  |  | Lower | Upper |
| Regional | 339.487 | 10.8676 | 318.187 | 360.787 |
| Distal-proximal | 303.944 | 10.7957 | 282.785 | 325.104 |
| Sham | 338.835 | 10.8740 | 317.522 | 360.147 |

The primary endpoint was examined with generalized linear model (GLM) and adjusting for weights, anesthesia duration and study sites.

**Table S3** **Difference(95% CI) of total sufentanil consumption among three groups**

| Group | Group | Means of Difference | Standard Error | *P* | 95% Confidence Interval | |
| --- | --- | --- | --- | --- | --- | --- |
|  |  |  |  |  | Lower | Upper |
| Distal-proximal | Regional | -35.542 | 15.3151 | 0.020 | -65.559 | -5.525 |
|  | Sham | -34.890 | 15.3286 | 0.023 | -64.934 | -4.847 |

The primary endpoint was examined with generalized linear model (GLM) and adjusting for weights, anesthesia duration and study sites.

**Table S4** **Subgroup analysis of total sufentanil consumption with GLM between distal-proximal group and regional group**

| Variable | Count | Percent | *p* | *p* for interaction | Relative  Risk(RR) | RR  Lower | RR  Upper |
| --- | --- | --- | --- | --- | --- | --- | --- |
| **Sex** | | | | 0.841 | | | |
| Female | 53 | 25.4 | 0.307 |  | 1.14 | 0.89 | 1.49 |
| Male | 156 | 74.6 | 0.058 |  | 1.12 | 1.00 | 1.25 |
| **Age** | | | | 0.517 | | | |
| < 65 | 131 | 62.7 | 0.162 |  | 1.09 | 0.96 | 1.25 |
| ≥ 65 | 78 | 37.3 | 0.058 |  | 1.20 | 1.00 | 1.43 |
| **Center** | | | | 0.27 | | | |
| i | 71 | 34 | 0.608 |  | 1.03 | 0.91 | 1.16 |
| ii | 74 | 35.4 | 0.238 |  | 1.11 | 0.94 | 1.32 |
| iii and iv | 32 | 15.3 | 0.408 |  | 1.13 | 0.85 | 1.48 |
| v | 32 | 15.3 | 0.106 |  | 1.39 | 0.95 | 2.14 |
| **NYHA** | | | | 0.632 | | | |
| I | 23 | 11 | 0.347 |  | 1.21 | 0.84 | 1.84 |
| II | 95 | 45.5 | 0.418 |  | 1.06 | 0.92 | 1.22 |
| III | 91 | 43.5 | 0.094 |  | 1.13 | 0.98 | 1.28 |
| **Clamped partial aorta** | | | | 0.049 | | | |
| No | 60 | 28.7 | 0.929 |  | 0.99 | 0.88 | 1.13 |
| Yes | 149 | 71.3 | 0.059 |  | 1.13 | 1.00 | 1.27 |
| **EuroSCORE I** | | | | 0.976 | | | |
| < 4 | 134 | 64.1 | 0.064 |  | 1.13 | 0.99 | 1.28 |
| ≥ 4 | 75 | 35.9 | 0.201 |  | 1.13 | 0.94 | 1.36 |

**Table S5** **Subgroup analysis of total sufentanil consumption with GLM between distal-proximal group and sham group**

| Variable | Count | Percent | *P* | *P* for interaction | Relative  Risk(RR) | RR  lower | RR  upper |
| --- | --- | --- | --- | --- | --- | --- | --- |
| **Gender** | | | | 0.238 | | | |
| Female | 40 | 19.1 | 0.828 |  | 0.98 | 0.79 | 1.20 |
| Male | 169 | 80.9 | 0.02 |  | 1.13 | 1.02 | 1.25 |
| **Age** | | | | 0.795 | | | |
| < 65 | 123 | 58.9 | 0.052 |  | 1.13 | 1.00 | 1.27 |
| ≥ 65 | 86 | 41.1 | 0.153 |  | 1.11 | 0.97 | 1.26 |
| **Centre** | | | | 0.084 | | | |
| i | 72 | 34.4 | 0.005 |  | 1.17 | 1.05 | 1.30 |
| ii | 71 | 34 | 0.607 |  | 1.04 | 0.91 | 1.20 |
| iii and iv | 31 | 14.8 | 0.63 |  | 0.96 | 0.80 | 1.15 |
| v | 35 | 16.7 | 0.159 |  | 1.31 | 0.91 | 1.94 |
| **NYHA** | | | | 0.081 | | | |
| I | 18 | 8.6 | 0.546 |  | 0.93 | 0.76 | 1.15 |
| II | 97 | 46.4 | 0.238 |  | 1.09 | 0.94 | 1.28 |
| III | 94 | 45 | 0.004 |  | 1.17 | 1.06 | 1.31 |
| **Clamped partial aorta** | | | | 0.099 | | | |
| No | 67 | 32.1 | 0.019 |  | 1.17 | 1.03 | 1.34 |
| Yes | 142 | 67.9 | 0.249 |  | 1.06 | 0.96 | 1.19 |
| **EuroSCORE I** | | | | 0.858 | | | |
| < 4 | 143 | 68.4 | 0.066 |  | 1.11 | 0.99 | 1.23 |
| ≥ 4 | 66 | 31.6 | 0.376 |  | 1.07 | 0.92 | 1.23 |
